# Active Obstacle Avoidance Trajectory Planning for Vehicles Based on Obstacle Potential Field and MPC in V2P Scenario

**DOI:** 10.3390/s23063248

**Published:** 2023-03-19

**Authors:** Ruoyu Pan, Lihua Jie, Xinyu Zhao, Honggang Wang, Jingfeng Yang, Jiwei Song

**Affiliations:** 1 School of Communications and Information Engineering and School of Artificial Intelligence, Xi’an University of Posts and Telecommunications, Xi’an 710121, China; 2 Guangzhou Institute of Industrial Intelligence, Guangzhou 511458, China; 3 China Electronics Standardization Institute, Beijing 100007, China

**Keywords:** V2P, artificial potential field, A*, MPC (model predictive control)

## Abstract

V2P (vehicle-to-pedestrian) communication can improve road traffic efficiency, solve traffic congestion, and improve traffic safety. It is an important direction for the development of smart transportation in the future. Existing V2P communication systems are limited to the early warning of vehicles and pedestrians, and do not plan the trajectory of vehicles to achieve active collision avoidance. In order to reduce the adverse effects on vehicle comfort and economy caused by switching the “stop–go” state, this paper uses a PF (particle filter) to preprocess GPS (Global Positioning System) data to solve the problem of poor positioning accuracy. An obstacle avoidance trajectory-planning algorithm that meets the needs of vehicle path planning is proposed, which considers the constraints of the road environment and pedestrian travel. The algorithm improves the obstacle repulsion model of the artificial potential field method, and combines it with the A* algorithm and model predictive control. At the same time, it controls the input and output based on the artificial potential field method and vehicle motion constraints, so as to obtain the planned trajectory of the vehicle’s active obstacle avoidance. The test results show that the vehicle trajectory planned by the algorithm is relatively smooth, and the acceleration and steering angle change ranges are small. Based on ensuring safety, stability, and comfort in vehicle driving, this trajectory can effectively prevent collisions between vehicles and pedestrians and improve traffic efficiency.

## 1. Introduction

In recent years, autonomous driving technology has undergone considerable improvement. Academic and industrial circles around the world are actively promoting practical commercial applications for this technology, injecting new vitality into the research of autonomous driving technology [1]. Self-driving cars rely on advanced intelligent control vehicle technology to reduce traffic accidents caused by driver errors, and even reduce the incidence of traffic accidents to zero. Autonomous driving technology must ensure accurate avoidance of pedestrians, so V2P is one of the core technologies in autonomous driving [2,3,4]. It notifies both people and vehicles by analyzing the key information of vehicles and pedestrians; this can prevent collisions between people and vehicles and effectively improve road traffic efficiency, in order to achieve intelligent transportation solutions for vehicle–road coordination.

V2P usually predicts whether there is a risk of collision between people and vehicles, and judges whether an alarm needs to be issued to warn pedestrians and vehicles. A V2X (vehicle-to-everything) collaborative system based on cellular and 802.11p radio is designed in [5], and the SafeNav Android application is proposed, in which the interface sets a determined collision area, and if multiple traffic participants enter the collision area, the color of the traffic participant changes to red and it produces a visual and audible alarm. In [3], a mobile application is proposed by Hussein et al. that supports pedestrians and vehicles. When the user interacts with their mobile phone, the screen is activated to detect the location coordinates of the mobile phone. The system calculates the potential collision point and judges whether collision is possible. This application, with its user-friendly operation, increases VRU (Vulnerable Road User)s’ visual situational awareness of locations near automatic- or manual-control vehicles. The above studies all needed to predict the degree of risk, and the vehicles needed to switch frequently to the “stop-go” state. The vehicles did not actively avoid pedestrians, and the degree of intelligence was not high, which may bring certain safety risks [6]. Trajectory planning can realize active obstacle avoidance for V2P vehicles [7]. In order to safely and quickly move a vehicle from its current location to the target location, it is necessary to design a robust trajectory planning strategy.

At present, classic trajectory planning includes rule-based methods [8,9], graph search methods [10,11,12], and numerical optimization methods [13]. The rule-based method aims to establish a behavior rule base based on vehicle driving rules and experience information, divide the state according to different environmental information, and determine the vehicle driving trajectory according to the rule logic. In [14], an autonomous vehicle control system based on the FSM (finite state machine) rule-based method was proposed by Sang-Hyeon Bae et al. The proposed vehicle system can handle six major urban environment driving situations. The system actively recognizes the real-time driving environment, formulates an action plan based on the finite state machine, and activates motion model control. The rule-based method can effectively deal with some typical driving scenarios defined in the rule base, but due to the limited number of added driving scenarios, it cannot handle driving scenarios that have not been added to the rule base; thus, it has certain limitations.

To solve the problem of limited driving scenarios, the graph search method is used for trajectory planning. Dijkstra, BFS (Best First Search), and A* are common graph search planning algorithms [15]. Dijkstra’s algorithm starts from the initial point where the object is located, visits the adjacent nodes of the initial point in the graph until it reaches the target point, and finally, outputs the shortest path; however, the algorithm runs for a long time and is not in real time. The BFS algorithm quickly guides the target point by introducing a heuristic function, thereby effectively improving the running speed of the algorithm. The heuristic function evaluates the cost from any node to the target node, and selects the node closest to the target instead of the node closest to the initial point. Therefore, there is no guarantee that the shortest path will be found. The A* algorithm combines the advantages of the heuristic algorithm, BFS, and the Dijkstra algorithm; not only can it find the shortest path, but in a simple static road environment, the algorithm running time is also as fast as BFS. This, it can meet the real-time requirements of vehicle trajectory planning [16]. The graph search method is suitable for situations where obstacles are stationary, while pedestrians cannot be considered static obstacles, similarly to roads, so they cannot be directly applied to V2P scenarios.

The artificial potential field method has become a classic algorithm in numerical optimization methods due to its simple principle, its small amount of calculation, and its high execution efficiency, and is widely used in robot trajectory planning [17]. The artificial potential field method has a small amount of calculation and fast calculation speed; thus, it meets the high real-time requirements of vehicle trajectory planning, and supports the independent design of the attraction potential field generated by the target position, and the repulsive potential field generated by obstacles, to meet the requirements of different scenarios. In [18], Lu et al. proposed a method to find a path by fitting the curve of the target point and the critical oscillation point, reducing the path jitter, and making the path smoother. Virtual repulsion was added by Azzabi A et al. [19] to improve the repulsion potential field function and solve the local optimum of the artificial potential field. However, since the direction of virtual repulsion is uncertain, it may cause new problems. The application of the above-mentioned artificial potential field method does not consider the vehicle’s motion constraints, so it cannot be directly applied to the vehicle trajectory planning problem.

Due to its ability to deal with system multi-constraint problems, the model predictive control (MPC) algorithm in numerical optimization is widely used in the field of automatic driving [20]. MPC establishes a mathematical model with a cost function and constraints. Constrained optimization problems can be solved using linear programming methods according to the cost and the complexity of the constraint function. The advantage is that the optimal trajectory is not constrained by a predefined pattern. Cost and constraint functions adapt to the driving environment with road geometry, boundaries, and obstacles. This adaptability makes the final trajectory highly adaptable to changes in the environment. A solution proposed by Mekala et al. was to use MPC to control speed for obstacle avoidance, so that the vehicle can accelerate and decelerate smoothly, but obstacles are represented as hard constraints in the optimization process; this may lead to the non-existence of feasible solutions in practice [21]. An iterative nonlinear model predictive control path planner using a point-mass vehicle model was proposed by Murillo et al., which considered vehicle dynamic constraints but not road boundaries [22].

This paper proposes an obstacle avoidance trajectory planning algorithm that meets the needs of vehicle path planning. First, pedestrians are ignored and only road boundaries are considered, and the A* algorithm is used to give the shortest path from the initial point of the vehicle to the target point as a reference path. Secondly, pedestrians are regarded as dynamic obstacles, and a linear prediction model is established on the basis of the vehicle model, which is used to predict the future state of the vehicle. Based on the artificial potential field method and the vehicle motion constraints, the input constraints and output variable constraints are jointly controlled to plan the vehicle’s trajectory. Finally, the trajectory planning algorithm proposed in this paper is verified via test verification.

The rest of this paper is organized as follows. Section 2 describes the preprocessing process of GPS data information. Section 3 introduces the proposed trajectory planning algorithm and gives the algorithmic framework to safely and efficiently solve the active obstacle avoidance problem in vehicles. Section 4 outlines the test verification and analyzes the results of the algorithm to verify the validity of the algorithm. Finally, the conclusions are given in Section 5.

## 2. Data Preprocessing

Due to the influence of noise in the transmission process, the GPS position information obtained by the actual system has incompleteness and uncertainty, which affects the accuracy of the position information. For noisy data, filters are usually used for preprocessing. The Kalman filter is only suitable for linear systems, and the actual system had different degrees of nonlinearity, so the nonlinear Kalman filter can be better for application in the filtering process of GPS data. In nonlinear filtering, the model is divided into a state model and a measurement model.

### 2.1. State Model

The basic state vector of the state model includes the GPS position coordinates, the velocity, and the error caused by noise in the propagation process. The first-order Gauss-Markov process was used to model the error as non-white noise. The state vector of the system can be given by expression (1).

(1) $$\begin{array}{l} x_{t}=[X_{t},\overset{\cdot }{X_{t}},Y_{t},\overset{\cdot }{Y_{t}},\varepsilon_{1t},\varepsilon_{2t},\ldots ,\varepsilon_{nt}]\\ x_{t+1}=f(x_{t}) \end{array}$$

where $\left(X_{t},Y_{t}\right)$ is the position coordinate component, $\left(\overset{\cdot }{X_{t}},\overset{\cdot }{Y_{t}}\right)$ is the velocity coordinate component, and $\varepsilon_{it}(i=1.2.\ldots ,n)$ is the error caused by the n-th noise source in the receiving process, so the state model of the system is obtained as follows:

(2) $$\begin{array}{l} X_{t+1}=X_{t}+T\overset{\cdot }{X_{t}}+\frac{T^{2}}{2}\delta \overset{\cdot \cdot }{X_{t}}\\ \overset{\cdot }{X_{t+1}}=\overset{\cdot }{X_{t}}+T\delta \overset{\cdot \cdot }{X_{t}}\\ Y_{t+1}=Y_{t}+T\overset{\cdot }{Y_{t}}+\frac{T^{2}}{2}\delta \overset{\cdot \cdot }{Y_{t}}\\ \overset{\cdot }{Y_{t+1}}=\overset{\cdot }{Y_{t}}+T\delta \overset{\cdot \cdot }{Y_{t}} \end{array}$$

where $\delta \overset{\cdot \cdot }{X_{t}},\delta \overset{\cdot \cdot }{Y_{t}}$ is the interference of other systems, T is the sampling period, and the error caused by noise in the propagation process is expressed using the first-order Gauss–Markov process as follows:

(3) $$\begin{array}{c} \varepsilon_{1(t+1)}=\alpha \varepsilon_{1t}+\beta \sigma_{1t}\\ \varepsilon_{2(t+1)}=\alpha \varepsilon_{2t}+\beta \sigma_{2t}\\ \vdots \\ \varepsilon_{n(t+1)}=\alpha \varepsilon_{nt}+\beta \sigma_{nt} \end{array}$$

$\sigma_{nt}$ is the error brought by the n-th noise source in the propagation process. This article assumes that n=2, and that these noises are non-correlated random white noise with a mean value of 0; the parameters α,β are, respectively,

$$\alpha =\frac{2\tau -1}{2\tau +1},\beta =\frac{2\tau }{2\tau +1}$$

where τ=100.

### 2.2. Measurement Model

Using Z(t) to represent the observed value of GPS position information at the time t, the measurement model is expressed as:

(4) $$Z_{t}=Hx_{t}+V_{t}$$

(5) $$\begin{array}{l} Z_{1t}=X_{t}+\varepsilon_{1t}+V(t)\\ Z_{2t}=Y_{t}+\varepsilon_{2t}+V(t) \end{array}$$

In the formula, it is assumed that V(t) is Gaussian white noise with variance $\sigma_{v}^{2}$ and a mean value of 0.

The system is discretized, and the obtained state model and measurement model are as follows:

(6) $$\begin{array}{l} x_{k+1}=f(x_{k})\\ Z_{k}=Hx_{k}+V_{k} \end{array}$$

where the subscript k represents the time.

## 3. Trajectory Planning

Trajectory planning was divided into path planning and speed planning. First, under the environment in which obstacles are ignored, the A* algorithm was used to construct the drivable area, and initial global planning of the path from the initial point to the target point was carried out to produce a reference path. Secondly, the artificial potential field method and MPC were combined for trajectory planning.

### 3.1. A* Path Planning

The Dijkstra, Best First Search (BFS), and A* algorithms are three typical path planning algorithms. Dijkstra’s algorithm is guaranteed to find the shortest path from the initial point to the goal point, but it runs slowly [23,24]. BFS runs faster than Dijkstra’s algorithm, but it cannot be guaranteed to find the shortest path [25]. The A* algorithm is a heuristic search algorithm that can perform global path planning in a static environment according to a defined evaluation function [17]. It responds quickly to the environment and searches the path directly, so it is widely used in path planning research. This algorithm can greatly reduce search time and improve path search efficiency while keeping the path as short as possible. Since the static road environment where the vehicle is driving is relatively simple, in simple cases, the A* algorithm runs as fast as BFS. Therefore, this paper uses the A* algorithm to plan the global path and generate a vehicle reference path.

The heuristic function of the A* algorithm contains information on the initial point and the target point at the same time, and the priority of each node is calculated using Formula (7):

(7) f(n)=g(n)+h(n)

where f(n) is the comprehensive priority of the node n. When selecting the next node to traverse, the node with the smallest f(n) value and the highest priority will be selected. g(n) is the cost of the node n from the initial point. h(n) is the heuristic function of the A* algorithm, and is the estimated cost of the node distance from the target point. This paper allowed the vehicle to move in eight directions (namely: front, rear, left, right, right front, right rear, left rear, and left front, corresponding to g(n) values of 1, 1, 1, 1, 1.5, 1.5, 1.5, and 1.5), and the Euclidean distance was chosen as the heuristic function.

### 3.2. Vehicle and Pedestrian Models

When using MPC for local trajectory planning, the motion states of vehicles and pedestrians are required. Therefore, the monorail vehicle kinematics model and the pedestrian (dynamic obstacle) model were established first, and were used to describe the state of the vehicle and pedestrian, respectively.

#### 3.2.1. Vehicle Model

**Figure 1:**
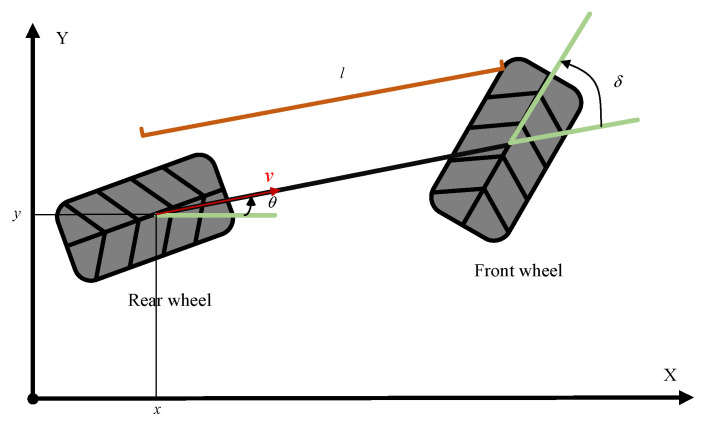
Schematic diagram of vehicle monorail model.

This paper mainly considers the plane motion of the vehicle, and used a single-track model to describe the vehicle, taking the rear wheel of the vehicle as a reference point, as shown in Figure 1. θ represents the direction of the speed of the vehicle, l represents the wheelbase, v represents the vehicle’s current speed, and δ represents the angle between the two speed directions at the front and rear of the vehicle.

The updated formula of each state quantity in the model is as follows:

(8) $$\begin{array}{l} x(t+dt)=x(t)+v(t)\cos(\theta )dt\\ y(t+dt)=y(t)+v(t)\sin(\theta )dt\\ \theta (t+dt)=\theta (t)+\frac{v}{l}\tan(\delta )dt\\ v(t+dt)=v(t)+adt \end{array}$$

where x and y are the longitudinal and lateral positions of the vehicle, and a is the acceleration of the vehicle.

#### 3.2.2. Pedestrian Model

The basic aim of the traditional artificial potential field method is to control the robot to find a collision-free path by constructing an artificial force field based on the attraction field of the target point and the repulsive force fields of the obstacles, and using the falling direction of the search potential function. The path planning of the robot does not need to consider the boundary and road environment, but for the vehicle, it needs to consider the constraints of the road, so the model construction is more complicated.

During the driving process of the vehicle, the most common dynamic obstacle is the pedestrian. In this paper, the pedestrian is abstracted as an obstacle point. Since the pedestrian is a dynamic obstacle, the position and velocity of the pedestrian should also be taken into account in the obstacle potential field. According to the space dynamics equation and the Lagrangian equation, the resultant force of the vehicle is the vector superposition of the repulsive force and the attraction force, and the direction of the resultant force is the moving direction of the vehicle. Under the action of the resultant force, the vehicle can bypass obstacles and reach the end point. The resultant force F can be expressed as:

(9) $$F=F_{a}+F_{r}$$

where $F_{a}$ is the attraction force generated by the target point on the controlled object, and $F_{r}$ is the repulsive force generated by the obstacle on the controlled object. Assuming there are n obstacle points, the attraction function $F_{a}$ and repulsion function $F_{r}$ are redefined as:

(10) $$F_{a}=\frac{1}{2}\times K_{a}\times \sqrt{{\left(x-g_{x}\right)}^{2}+{\left(y-g_{y}\right)}^{2}}$$

(11) $$F_{r}=\left\{\begin{array}{c} \frac{1}{2}\times K_{r}\times e^{-\sum_{i=1}^{n}\sqrt{{\left(x-O_{x_{i}}\right)}^{2}+{\left(y-O_{y_{i}}\right)}^{2}}}\\ 0,d>d_{r} \end{array}\right.,0<d<d_{r}$$

where (x,y) are the position coordinates of the vehicle at any time, $\left(g_{x},g_{y}\right)$ are the coordinates of the end position of the vehicle, $K_{a}$ is the gravitational potential energy gain coefficient, and d is the Euclidean distance between the vehicle and the nearest obstacle. $d_{r}$ is the radius of the obstacle repulsion field. When the distance between the vehicle and the obstacle is less than $d_{r}$, the vehicle will be affected by the repulsion. $K_{r}$ is the repulsive potential energy gain coefficient, $\left(O_{x_{i}},O_{y_{i}}\right)$ are the obstacle coordinates, and $O_{x_{i}}=\left[x_{i},v_{x_{i}}\right]$, $O_{y_{i}}=\left[x_{i},v_{y_{i}}\right]$ is the combination of position and velocity.

### 3.3. MPC Trajectory Planning

The MPC method is a numerical optimization method. According to the model and the current state quantity output, the deviation between the predicted trajectory and the expected trajectory is calculated, and control quantity input and output constraints are imposed to ensure that the vehicle can meet the corresponding motion constraints and avoid collisions. In the trajectory planning process, the future behavior of the vehicle needs to be predicted within the specified forecast horizon, and the control input at the next moment is calculated by minimizing the error between the predictor and the reference point under various constraints. On the basis of Formula (8), the linear prediction model of the current sampling moment is established as follows:

(12) $$\begin{array}{l} \dot{x}(t+dt)=v\cos(\phi )-v\theta \sin(\phi )\\ \dot{y}(t+dt)=v\theta \cos(\phi )+v\sin(\phi )\\ \dot{\phi }(t+dt)=\frac{v\tan(\delta )}{l}+\frac{v\delta \tan(\delta^{2}+1)}{l}\\ \phi =\theta +\delta \end{array}$$

Since the controlled system in MPC usually adopts a discrete state space model, it is necessary to establish a state space expression under a linear discrete time system, and expand the state space variable $x_{s}(k)$ and the control variable u(k) into a new state variable $x_{s}(k+1)$. The new state space expression is as follows:

(13) $$\begin{array}{l} x_{s}(k+1)=Ax_{s}(k)+Bu(k)+C\\ y_{o}(k)=Dx(k) \end{array}$$

where $x_{s}(k)\in {\mathbb{R}}^{4}$, $u(k)\in {\mathbb{R}}^{2}$, and $y_{o}(k)\in {\mathbb{R}}^{2}$ are output variables. $x_{s}(k),y_{o}(k)$ are all related to the four identical variables, and Formula (14) lists the expression for $x_{s}(k)$.

(14) $$\begin{array}{l} x_{s}(k)={\left[x(k),y(k),\phi (k),v(k)\right]}^{\prime }\\ u(k)={\left[a(k),\delta (k)\right]}^{\prime }\\ \dot{v}(k)=a(k) \end{array}$$

(15) $$\begin{array}{l} A=\left[\begin{array}{cccc} 1 & 0 & -v\sin(\theta ) & \cos(\theta )\\ 0 & 1 & v\cos(\theta ) & \sin(\theta )\\ 0 & 0 & 1 & \frac{\tan(\delta )}{l}\\ 0 & 0 & 0 & 1 \end{array}\right]\\ B=\left[\begin{array}{cc} 0 & 0\\ 0 & 0\\ 0 & \frac{v}{l\cos^{2}(\delta )}\\ 1 & 0 \end{array}\right]\\ C=\left[v\theta \begin{array}{cccc} \sin(\theta ) & -v\theta \cos(\theta ) & -v\frac{\delta }{l\cos^{2}(\delta )} & 0 \end{array}\right]\\ D=\left[\begin{array}{cccc} 1 & 0 & 0 & 0\\ 0 & 1 & 0 & 0\\ 0 & 0 & 1 & 0\\ 0 & 0 & 0 & 1 \end{array}\right] \end{array}$$

Based on the state space model for predicting the future dynamics of the system, Formula (13) is rewritten as an incremental model as follows:

(16) $$\begin{array}{l} \Delta x_{s}(k+1)=A\Delta x_{s}(k)+B\Delta u(k)\\ \Delta y_{o}(k)=C\Delta x(k) \end{array}$$

where

(17) $$\begin{array}{l} \Delta x_{s}(k)=x_{s}(k)-x_{s}(k-1)\\ \Delta u(k)=u(k)-u(k-1) \end{array}$$

The prediction range of the MPC-based upper decision controller is set to $N_{p}$ and the control range is set to $N_{c}$. In addition, $N_{p}<N_{c}$. Assuming that the current moment is k, k>0. Using the current state information to define the input vector and predicted output vector of the system in the future $N_{c}$, the steps are as follows:

(18) $$U(k)\overset{def}{=}\left[\begin{array}{c} u(k)\\ u(k+1)\\ \ldots \\ u(k+N_{c}-1) \end{array}\right]$$

(19) $$Y_{N_{p}}(k+1|k)\overset{def}{=}\left[\begin{array}{c} y_{o}(k+1|k)\\ y_{o}(k+2|k)\\ \ldots \\ y_{o}(k+N_{p}|k) \end{array}\right]$$

The output vector prediction equation for the next $N_{p}$ steps is as follows:

(20) $$Y_{N_{p}}(k+1|k)=S_{x}x_{s}(k)+S_{u}U(k)$$

where

(21) $$S_{x}=[CA,CA^{2}\ldots CA^{N_{p}}]$$

(22) $$S_{u}=\left[\begin{array}{cccc} CB & 0 & \cdots & 0\\ CAB & CB & \cdots & 0\\ \vdots & \vdots & \ddots & \vdots \\ CA^{N_{p}-1}B & CA^{N_{p}-2}B & \cdots & \sum_{i=1}^{N_{P}-N_{c}+1}CA^{i}B \end{array}\right]$$

In order to ensure that the predicted output variable is as close as possible to the reference trajectory, that is, that the vehicle uses the shortest path as much as possible, the objective function for constructing the optimization solution is as follows:

(23) $$\min J=K\sum_{k=0}^{N_{p}}∥Y_{N_{p}}(k+1|k)-R(k+1){∥}_{Q}^{2}+K_{u}∥\Delta U(k){∥}_{R}^{2}+\sum_{k=0}^{N_{p}}F_{r}(k+1|k)+K_{h}h^{2}$$

In the formula, $K_{h}$ is the weight coefficient, h is the relaxation factor, Q,R are the weighted matrices of the output error and control input, respectively, and R(k+1) is the reference path, which is the path obtained by the A algorithm. The first item of the objective function represents the planning trajectory, and the reference trajectory should be as close as possible to ensure the shortest planned trajectory. The second item aims to control the incremental size to ensure that there will be no drastic changes in speed, heading angle, or acceleration during the operation of the vehicle, and to ensure the stability and comfort of the vehicle to a certain extent. The third item represents the potential energy value of the vehicle in the obstacle potential field, which ensures that the vehicle can effectively avoid dynamic obstacles. The fourth term is the relaxation factor, which can enhance the solution of the feasible domain, so as to ensure that there is an optimal solution to the programming problem.

To ensure the safety of vehicle obstacle avoidance and reduce the influence of the maneuvering process on the comfort of the vehicle, the control process needs to take into account the corresponding constraints. Through these constraints, the vehicle can avoid accelerating or decelerating too quickly, thereby ensuring driving safety and comfort, and the control input must meet the following constraints [26,27]:

(24) $$\left\{\begin{array}{l} -8{\;m/s}^{2}\leq a\leq 3{\;m/s}^{2},the\;pedestrian\;was\;not\;detected\\ -2{\;m/s}^{2}\leq a\leq 1{\;m/s}^{2},the\;pedestrian\;was\;detected\\ -\pi /4\leq \delta \leq \pi /4\\ 0\;m/s\leq v\leq 16.7\;m/s\\ v_{ref}=12\;m/s \end{array}\right.$$

where $v_{ref}$ is the best reference speed. Speeds of 12 m/s and 16 m/s were chosen because they correspond to typical speeds of 40 m/h and 60 m/h.

### 3.4. Algorithm Framework Description

**Figure 2:**
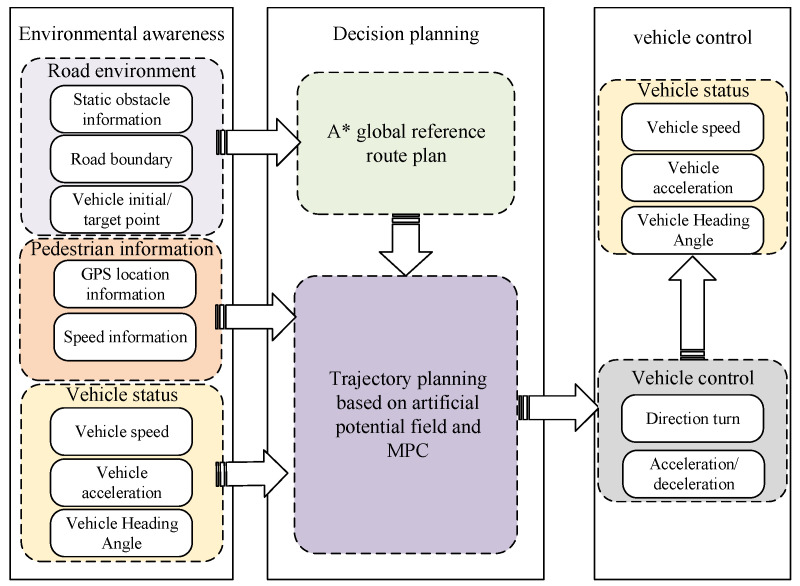
The framework of the trajectory planning algorithm system proposed in this paper.

Figure 2 describes the proposed algorithm framework. The algorithm takes the road environment, pedestrian trajectory, and vehicle running state as inputs, and combines the A* algorithm, the artificial potential field method, and MPC trajectory planning to finally output the vehicle control state and guide the vehicle to avoid obstacles. First, according to the initial point and target point of the vehicle, combined with the road environment, the A* algorithm is used to plan a global path, which will function as the reference path. Secondly, based on the global path, PF processes the position information of pedestrians and vehicles, and uses the artificial potential field method and MPC algorithm to plan a trajectory that satisfies the obstacle model and vehicle motion constraints.

## 4. Test Verification and Analysis of Results

### 4.1. Test Environment

The test required pedestrians and vehicles to be equipped with GPS to determine their position, speed, and direction. The pedestrian terminal included GPS and LoRa modules, and the GPS working frequency was 1 HZ. MCU (Microcontroller Unit) adopted an STM32 chip, and the radio frequency module was the LoRa RFM98 chip.

**Figure 3:**
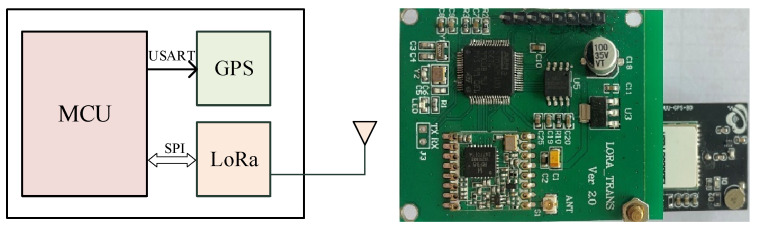
Pedestrian terminal (the hardware frame diagram is on the **left**, and the physical hardware diagram is on the **right**).

**Figure 4:**
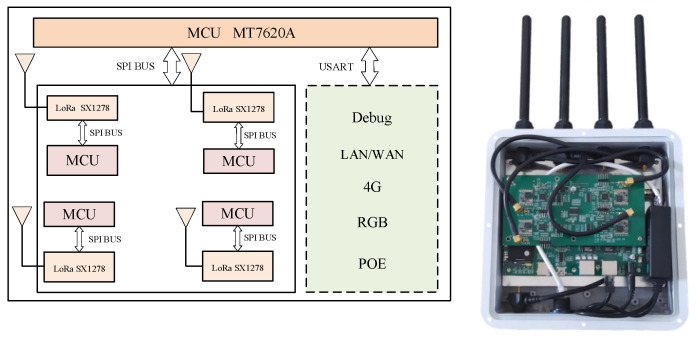
Gateway (the hardware frame diagram is on the **left** and the physical hardware diagram is on the **right**).

The test used the LoRa wireless transmission system, which consisted of gateways and terminals. The gateway was responsible for receiving and processing messages sent by the terminal. Figure 3 presents a hardware frame diagram and hardware physical diagram of the pedestrian terminal, in which the GPS module and the MCU perform one-way communication through the USART (Universal Synchronous/Asynchronous Receiver/Transmitter), and the LoRa module performs two-way communication with the MCU through the SPI (Serial Peripheral Interface) bus. Figure 4 is a hardware frame diagram of the gateway and its corresponding physical diagram. MT7620A is the main control chip of the gateway. The chip can meet the design requirements of the smart gateway in terms of performance, and its price is lower than that of similar chips; thus, it conforms to the low cost requirements of the LoRa network. It consists of four groups of the same modules, which can support simultaneous communication with different frequency bandwidths. Among them, LoRa communicates with the MCU through the SPI bus in a two-way manner. The gateway software runs on the OpenWRT operating system. The LoRa radio frequency module and its MCU in the gateway are the same as those of the terminal. The gateway uses a POE power supply, and an RGB indicator light indicates its working status. It possesses a single debugging serial port, an Ethernet interface LAN/WAN, and 4G. The module interface has dial-up Internet access.

The gateway was located on the roof of the No. 3 experimental building (15 m in height) of Xi’an University of Posts and Telecommunications. Pedestrians wearing V2P pedestrian terminals walked at a constant speed outside the teaching building of the Chang’an Campus of Xi’an University of Posts and Telecommunications.

### 4.2. Analysis of Results

**Figure 5:**
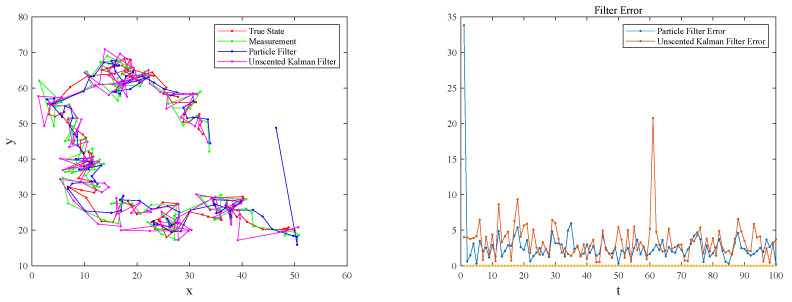
Analysis of filtering error of PF and UKF algorithms (the **left** picture shows the trajectory generated via filtering after collecting GPS position information, and the track generated by the actual position information. The **right** picture shows the error between the data processed by the filter and the real data).

Using the state model and measurement model in Section 2.1, pedestrian terminals and Superstar GPS OEM GPS receivers were used to collect pedestrian position information at the same time. The sampling interval was 1 s. The experiment collected 1100 sets of data, which were divided into eleven groups. UKF and PFs were used to filter The GPS position information, and the number of particles selected by the PFs was 100. A group was randomly selected, and the trajectory and filtering errors obtained are shown in Figure 5; the experimental results of the other ten groups are consistent with this group.

EKF, UKF, and PF are commonly used filters in nonlinear systems. EKF revolves around its current state through Taylor expansion, discarding high-order terms, linearizing the system under study, and solving it as a linearized system; the result has large deviation. The UKF algorithm uses UT transformation to obtain a Sigma point set, and uses a small number of points to approximate the state distribution, which better describes the nonlinear system, so the filter converges to the correct solution more quickly. However, UKF is only applicable to the standard Kalman filter system under linear assumption (under the linear and Gaussian assumption of the system). When the linearity of the system is not high, the effect of UKF filtering is not good. PF is a parameterless filter that does not have a specific function that enables it to obtain posterior results, and the Monte Carlo method generates a large number of random particles to approximate the posterior distribution of the state and achieve state estimation. In the process of prediction and updating, the PF algorithm only updates the particles in it, and then, obtains a state estimation via weighting and summing. It does not need to calculate the posterior covariance of the state, so it can approximate any system’s state distribution. In this paper, UKF and PF were selected to filter the GPS position information. It can be seen from Figure 5 that the trajectory filtered by PF is closer to the real trajectory. For stability, therefore, the PF filter was used to preprocess the data and improve the positioning accuracy.

**Figure 6:**
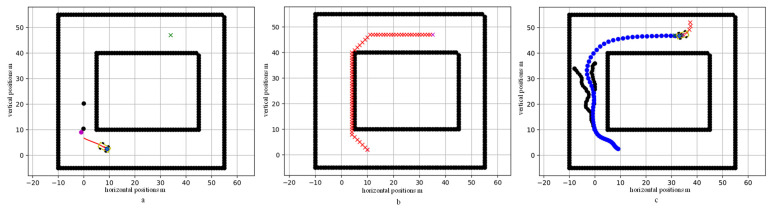
The trajectory generated by the proposed algorithm (the unit in the figure is m). (**a**) The initial state of the test scene, where the green mark at (35,47) is the target point, and the vehicle is at (10,2) as the initial coordinate point. (0,10) and (0,20) are the initial points of the two pedestrians. (**b**) The reference global path planned using the A* algorithm. (**c**) The path diagram planned using the proposed algorithm.

To verify the effectiveness of the proposed trajectory planning algorithm, the algorithm was tested and verified in this paper. Two pedestrians wearing pedestrian terminals kept moving forward at a constant speed, and the pedestrian speed was 1 m/s. After collecting the GPS position information of pedestrians, this paper modeled the pedestrian driving environment, assuming that the vehicle length was 4.5 m, the width was 2 m, the tire diameter was 0.6 m, the width was 0.25 m, and the front and rear wheelbases, and the rear wheelbases, were 1.5 m. As shown in Figure 6, the vehicle was modeled to a given size. The initial point was (10,2), the target point was (35,47), the best reference speed was given as 12 m/s, the pedestrian was located outside the line of sight of the driver, and the trajectory was uncertain. (0,10). The initial points of the two pedestrians were (0,20). After preprocessing the GPS data using PF, the positions of the vehicle and the pedestrian were determined and are shown in Figure 6a. The two black rectangles in the figure jointly denote the road boundary, which is regarded as a static obstacle, and pedestrians are dynamic obstacles relative to vehicles.

According to the initial point and target point of the given vehicle, we first used the A* algorithm to plan an optimal global reference path. The output trajectory of the A* algorithm is given by the discrete points in the predicted time domain, which are discrete trajectory points. Due to the kinematic constraints of the vehicle, if the vehicle position is required to be continuous, the yaw angle continuity requires the curve to be first-order continuous, and the acceleration constraint requires the curve to be second-order continuous; then, the planned path should satisfy the second-order continuity of the curve. Upon integrating the requirements of path planning and calculation cost, the cubic spline curve was selected to smooth the path. Figure 6b shows the fitting results of the planned path using the cubic spline curve.

**Figure 7:**
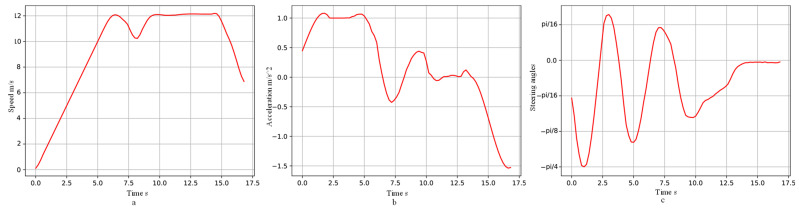
Changes in vehicle speed, acceleration, and heading angle during the trajectory planning process of the proposed algorithm. (**a**) The variation of velocity. (**b**) The variation of acceleration. (**c**) The variation of heading angle.

After testing and verification, the vehicle could avoid pedestrians, and the trajectory planned by the algorithm is shown in Figure 6c. The blue part is the vehicle trajectory, and the black part is the trajectory of the two pedestrians. The obstacle detection range was set to the front wheel of the vehicle as the reference point, within a circle with a radius of 5 m. The speed, acceleration, and heading angle of the vehicle during obstacle avoidance are shown in Figure 7. The vehicle accelerates as closely as possible to the recommended speed of 12 m/s. It stops accelerating to avoid the first pedestrian at the sixth second, and then, decelerates to avoid the second pedestrian, with complete collision avoidance, at the tenth second; finally, it decelerates within the speed limit to reach the target point. The vehicle heading angle changes smoothly within (−pi/4,pi/8).

**Figure 8:**
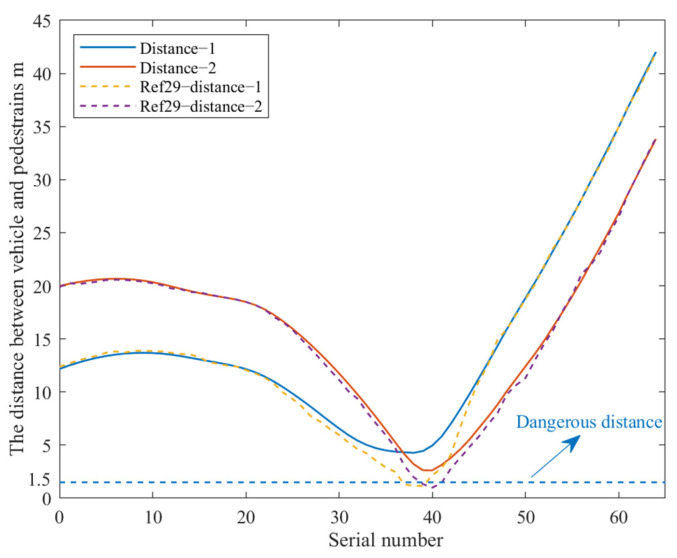
The distance between the vehicle and the pedestrian changes during obstacle avoidance. (the ordinate is the distance between the vehicle and the pedestrian, the unit is m, and the abscissa is the number of sequences).

In the case of the vehicle’s avoidance of pedestrians, the hazard distance between the vehicle and the pedestrian was set to 1.5 m [28]. In this paper, the closest distance between the vehicle and the pedestrian was used as the performance index to evaluate the proposed algorithm. In the process of algorithm execution, the distance between the vehicle and the two pedestrians was counted, and the result is shown in Figure 8; the ordinate is the distance between the vehicle and the pedestrian, the unit is m, and the abscissa is the number of sequences. Among them, the distance between the vehicle and the two pedestrians in the algorithm proposed in this paper was two solid lines. “Distance–1” in the legend indicates the distance between the vehicle and the first pedestrian during the operation of the proposed algorithm; “Distance–2” in the legend indicates the distance between the vehicle and the first pedestrian during the operation of the proposed algorithm; and “Ref. [29]–distance–1” in the legend represents the distance between the vehicle and the first pedestrian during the operation of the algorithm proposed by Ref. [29]. It can be seen from Figure 8 that the algorithm proposed in this paper has good obstacle avoidance performance, and the closest distance from pedestrians is 2.7 m, while the algorithm proposed by Ref. [29] is within the danger-range distance of pedestrians. Therefore, the algorithm in this paper has better performance and higher safety, and thus, has reference value.

## 5. Conclusions

In order to achieve the goals of vehicles actively avoiding pedestrians, and safely and reliably improving traffic efficiency, this paper proposes a trajectory planning algorithm that combines the A* algorithm, the artificial potential field method, and MPC; we added the dynamic obstacle potential field into the objective function of the MPC controller, so as to guide the vehicle to avoid obstacles and avoid of human–vehicle collision. The test results show that the planning algorithm proposed in this paper can plan the trajectory of vehicles while keeping a safe distance from pedestrians, to actively avoid collisions and improve traffic safety. The trajectory planned by this algorithm is relatively smooth and conforms to the motion constraints of the vehicle. During vehicle driving, the acceleration change is within the range of $\left(-1.6m/s^{2},1.2m/s^{2}\right)$, acceleration during vehicle obstacle avoidance is within the range of $\left(-1.6m/s^{2},0.4m/s^{2}\right)$, the heading angle change is in the range of (−pi/4,pi/8), and the vehicle acceleration and steering angle change ranges are small, ensuring steering stability and riding comfort.

## Data Availability

No applicable.
